# Congenital Isolation of a Common Carotid Artery: A Review of Cases, Phenotypes, and Embryology of a Rare Anomaly of the Aortic Arch System

**DOI:** 10.1007/s00246-025-04133-8

**Published:** 2025-12-13

**Authors:** Jörg Männer

**Affiliations:** Group Cardio-Embryology, Institute for Anatomy and Cell Biology, UMG, Georg August University Göttingen, Kreuzbergring 36, 37075 Göttingen, Germany

**Keywords:** Malformations, Aortic arch system, Isolated common carotid artery, Right aortic arch, Embryology, Aortic sac malseptation

## Abstract

Rare malformations of the aortic arch system may be of minor relevance for general medical practice. Their abnormal arterial patterns, however, sometimes can represent a challenge not only for experts in cardiovascular medicine but also for the validity of well-established concepts traditionally used to explain the embryology of normal and abnormal patterning of the aortic arch system. The present article provides a review of (1) the pathomorphological features, (2) associated cardiac and extracardiac malformations, and (3) embryology of a very rare malformation of the aortic arch system, called *isolation of a common carotid artery*. It is shown that this diagnosis does not characterize a single pathomorphological entity. Among 28 previously documented cases we can distinguish two different subsets. One of these subsets is of general interest since it challenges the traditional concept of the embryology of congenital malformations of the aortic arch system. It is shown that malseptation of the embryonic aortic sac and cardiac outflow tract may play a fundamental role in the pathogenesis of this malformation.

## Introduction

The diagnosis *isolation of a common carotid artery* characterizes very rare congenital anomalies of the aortic arch system where one of the two common carotid arteries (CCA) does not take its origin from the aortic arch or its main branches but takes its origin from the pulmonary trunk or from the left or right pulmonary artery. Up to now, only 28 cases were described in the literature. The majority of which were cases of isolation of the left common carotid artery (LCCA). Only two cases showed isolation of the right common carotid artery (RCCA).

For the present review article, the author has analyzed the pathomorphological features of all previously published cases of isolation of a CCA. It is shown that this anomaly of the aortic arch system is characterized by a triad of regularly associated anatomical features: (1) Abnormal origin of the isolated artery from a pulmonary artery (see above). (2) Presence of an aortic arch whose sidedness is contralateral to the sidedness of the isolated artery. (3) Presence of an aberrant subclavian artery. Thus, isolation of the LCCA is regularly associated with a right aortic arch and an aberrant left subclavian artery, while isolation of the RCCA is regularly associated with a left aortic arch and an aberrant right subclavian artery.

The above-mentioned triad of pathomorphological features suggests that the diagnosis *isolation of a CCA* may characterize a single pathomorphogenetic entity. However, the author’s analysis of the characteristics of the previously documented 28 cases shows that this is not the case. Among the previously published cases we can distinguish two different subsets that differ from each other with regard to phenotype, associated malformations, and embryology. The present article provides a review of the main pathomorphological features found in these cases, their associated cardiac and extracardiac malformations, and their embryology. This rare vascular malformation is of general interest since it challenges the traditional concept of the embryology of congenital malformations of the aortic arch system.

## Phenotypes of Isolated CCAs

The total number of previously published reports on human patients with isolated CCA is 30. The true number of documented cases, however, is 28. Two reports have described the same case [1, 2] and one case [3] turned out to be a case of right aortic arch with aberrant left brachiocephalic (innominate) artery. Among the previously published reports, a detailed description of the pathomorphological phenotype is provided in 27 cases (Tables 1 and 2). For a single case of isolated LCCA only the above-mentioned triad is documented [30] so that its phenotype cannot be assigned to one of the above-mentioned subsets.

**Table 1:** Characteristics of cases of isolated CCA with distal origin from the pulmonary trunk (bifurcation) or from a pulmonary artery

Report	Age at diagnosis	Sex	ICCA sidedness	ICCA origin	ICCA Condition of initial segment	Separate ipsilateral arterial duct	Contra-lateral arterial duct	Ao- arch sidedness	Associated vascular anomalies	Associated CHDs	Associated malformations/syndromes
Gelb et al. [4]	2 y	F	RCCA	RPA	open	–	patent	Left	ARSA	?	DiGeorge
Romberg et al. [5]	14 m	M	RCCA	RPA	open	–	–	Left	ARSA	VSD musc	22q11-del
Kanyo et al. [6]	2 y	?	LCCA	LPA	open	–	–	Right	ALSA	Partial APVR	22q11.2-del
Aboelsaud et al. [7]	Neonate (1 d)	F	LCCA	LPA	open	–	patent	Right	ALSA	Interrupt-RAA, AoV-hypoplasia, VSD	DiGeorge, 22q11.2-del
Liu et al. [8]	6 y	M	LCCA	LPA	open	–	–	Right	ALSA	–	–
Gao et al. [1]; Liu et al. [2]	Neonate (8 d)	F	LCCA	PT distal	open		patent	Right	ALSA	ASD, VSD pm	CHARGE
Auquilla et al. [9]	1 y	F	LCCA	PT distal	open	–	–	Right	ALSA	ASD, VSD, PVS	Holt-Oram
Vignaroli et al. [10]	2 m	M	LCCA	LPA	open	–	–	Right	ALSA	VSD sub-ao	22q11.2-del
Murayama et al. [11]	Neonate	?	LCCA	PT distal	open	–	patent	Right	ALSA	DORV, VSD	DiGeorge
Matsuo et al. [12]	Neonate	F	LCCA	PT distal	spontaneous closure	–	–	Right	ALSA	ToF, PVS, bicuspid PV	Ano-vulvar fistula
Cohen et al. [13]	Neonate	F	LCCA	PT distal	open	–	–	Right	ALSA	PV-atresia with intact VS	CHARGE
Nguyen [14]	15 y	F	LCCA	PT distal	open	–	–	Right	ALSA	–	–
Osakwe et al. [15]	Neonate	M	LCCA	PT distal	open	–	–	Right	ALSA	Ebstein anomaly, VSD, fenestrated AS, bicuspid AoV	CHARGE
Ahmadi et al. [16]	Neonate (3 w)	F	LCCA	PT distal	open	–	patent	Right	ALSA	AoV-atresia, Ao-hypoplasia	Abnormal facial structures
Tomek et al. [17]	Neonate (3 w)	M	LCCA	PT distal	open	–	patent	Right	ALSA	–	CHARGE
Oppido et al. [18]	Neonate	F	LCCA	LPA	open	–	patent	Right	ALSA	ToF	DiGeorge
Huang & Wu [19]	Neonate (premature)	F	LCCA	LPA	open	–	–	Right	ALSA	ToF, PT-atresia	DiGeorge
Ghalili et al. [20]	Neonate (6 d)	F	LCCA	PT distal	open	–	patent	Right	ALSA	Secundum ASD	CHARGE
Tozzi et al. [21]	3 y	?	LCCA	LPA	open	–	–	Right	ALSA	ToF	–
Fong & Venables [22]	5 y	F	LCCA	LPA	open	–	–	Right	ALSA	Primum ASD	Dwarfism

Ao = aorta; AoV = aortic valve; ALSA = aberrant left sbclavian artery; APVR = anomalous pulmonary venous return; ARSA = aberrant right subclavian artery; AS = atrial septum; ASD = atrial septal defect; CHDs = congenital heart defects; d = days; DORV = double outlet right ventricle; F = female; ICCA = isolated common carotid artery; interrup = interrupted; LCCA = left common carotid artery; LPA = left pulmonary artery; m = months; M = male; musc = muscular; pm = perimembranous; PT = pulmonary trunk; PT distal = distal end of pulmonary trunk (bifurcation); PV = pulmonary valve; PVS = pulmonary valve stenosis; RAA = right aortic arch; RCCA = right common carotid artery; RPA = right pulmonary artery; sub-ao = subaortic; ToF = Tetralogy of Fallot; VS = ventricular septum; VSD = ventricular septal defect; y = years. Note that the presence of a contralateral arterial duct was diagnosed by clinical imaging techniques (echocardiography, angiography), which facilitate diagnosis of an open duct but do not facilitate imaging of an arterial ligament or closed duct

**Table 2:** Characteristics of cases of isolated CCA with proximal origin from the pulmonary trunk

Report	Age at diagnosis	Sex	ICCA sidedness	ICCA origin	ICCA Condition of initial segment	Separate ipsilateral arterial duct	Contra-lateral arterial duct	Ao- arch sidedness	Associated vascular anomalies	Associated CHDs	Associated malformations/syndromes
Alenizi et al. [23]	1 st decade	F	LCCA	PT prox right	open	ligated	–	Right	ALSA	–	–
Sachdeva et al. [24]	5 y	M	LCCA	PT prox right	open, elastic artery, *no* ductal tissue	Ligament, LPA	–	Right	ALSA	–	Prominent forehead, depressed nasal bridges, hypertelorism
Juergensen et al. [25]	Neonate	M	LCCA	PT prox right	open	patent, ductal tissue	–	Right	ALSA	–	–
Sinha et al. [26]	6 y	M	LCCA	PT prox right	open	Ligament	–	Right	ALSA	–	–
Joshi et al. [27]	18 y	M	LCCA	PT prox right	open	Ligament	–	Right	ALSA	–	–
Fouilloux et al. [28]	3 y	M	LCCA	PT prox right	open, *no* ductal tissue	Ligament, LPA	–	Right	ALSA	–	–
Kaushik et al. [29]	10 m	M	LCCA	PT prox right	open	Ligament	–	Right	ALSA	–	Mild left hemifacial microsomia

PT prox right = right aspect of pulmonary trunk proximal to bifurcation; other abbreviations as used in Table 1. Note that with regard to an ipsilateral arterial duct, some information (e.g. presence and localization of an arterial ligament; histological structure of the initial segment of the isolated LCCA) was obtained in consequence of surgical correction of the arterial malformation

**Fig. 1:**
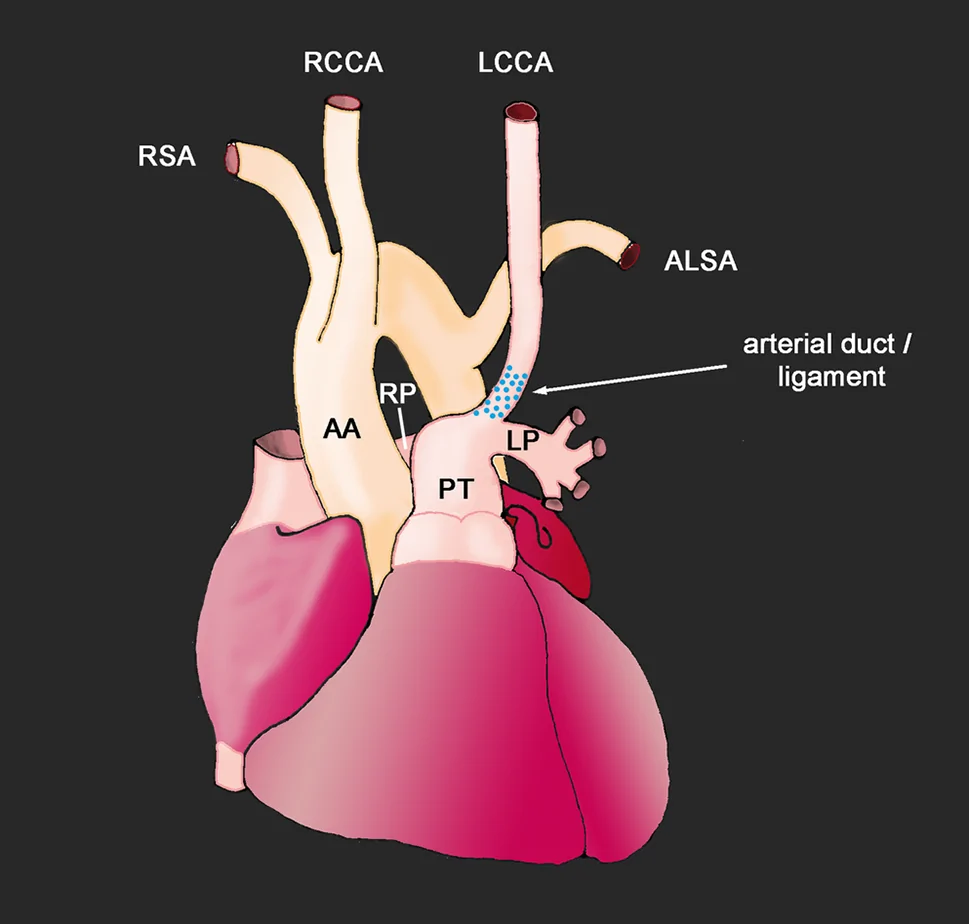
This drawing depicts the phenotype of isolation of the left common carotid artery with distal origin from the pulmonary trunk or from the left pulmonary artery. The isolated artery arises from the normal site of origin of the left arterial duct. Its initial segment thus is interpreted as derivative of the arterial duct (marked by blue dots). AA = ascending aorta; ALSA = aberrant left subclavian artery; LCCA = left common carotid artery; LP = left pulmonary artery; PT = pulmonary trunk; RCCA = right common carotid artery; RP = right pulmonary artery; RSA = right subclavian artery. Based on 3D CT image data from previous case reports [8, 9, 11]

The phenotype of the first subset of cases (Fig. 1; Table 1) may be named *isolated CCA with distal origin from the pulmonary trunk or pulmonary artery*. It is found in the majority of previously published cases (20/28). The isolated CCA runs outside of the pericardial cavity and takes its origin from the roof of the bifurcation site of the pulmonary trunk or from the left (isolated LCCA) or right pulmonary artery (isolated RCCA). These sites correspond to the sites of normal origin of the left and right arterial ducts, respectively. Consequently, the initial segment of this type of isolated CCA is generally interpreted as the ipsilateral arterial duct and this interpretation is supported by the fact that this segment sometimes undergoes postnatal narrowing or closure [12].

**Fig. 2:**
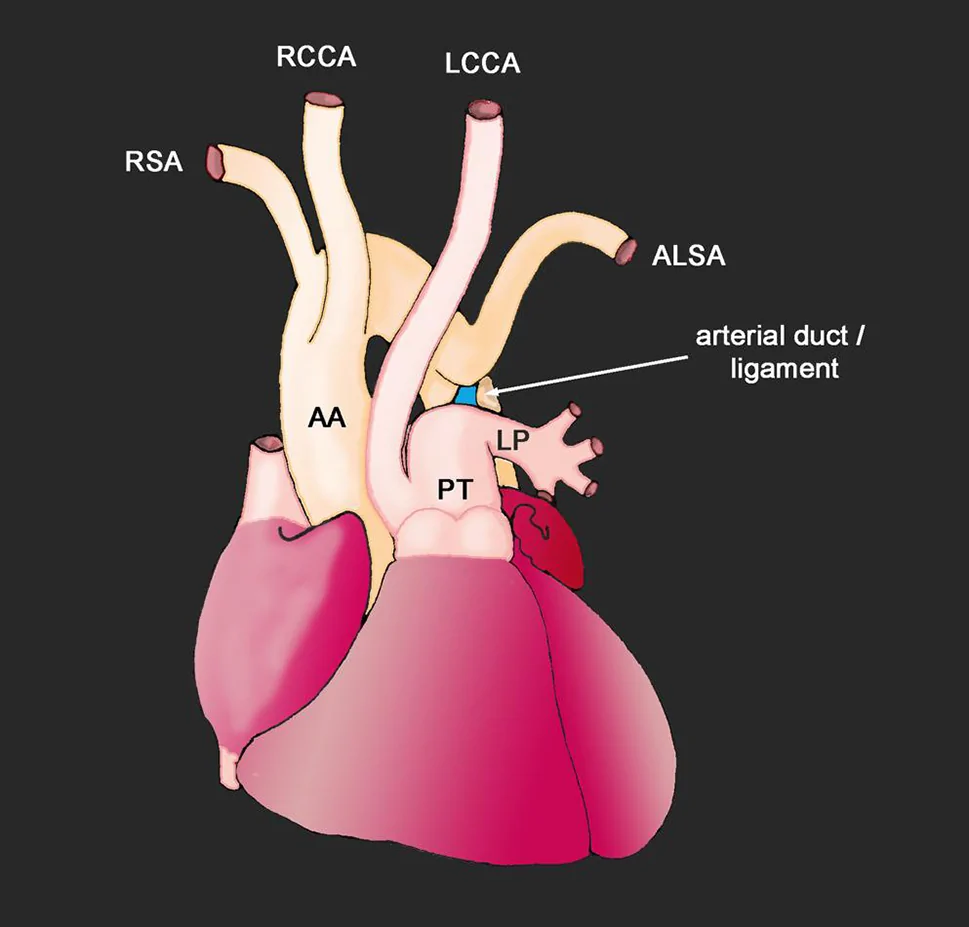
This drawing depicts the phenotype of isolation of the left common carotid artery with proximal origin from the pulmonary trunk. The isolated artery arises from the right aspect of the proximal main pulmonary artery close to the sinutubular junction. A remnant of the left arterial duct is found at the normal site of origin of the duct (marked by blue color). The initial segment of the isolated artery thus cannot be interpreted as derivative of the arterial duct. Abbreviations as used in Fig. 1. Note that the right pulmonary artery is not visable since it lies behind the initial segment of the isolated artery. Based on 3D CT image data from previous case reports [23, 24, 26, 29]

The phenotype of the second subset of cases (Fig. 2; Table 2) may be named *isolated CCA with proximal origin from the pulmonary trunk*. It is found only in a quarter of previously published cases (7/28), all of which are cases of isolated LCCA. Here, the isolated artery has a relatively long initial segment that arises from the right aspect of the proximal portion of the pulmonary trunk, mostly close to the sinutubular junction. This segment has an intrapericardial course along the left side of the ascending aorta where portions of its wall can be fused to the wall of the aorta [29]. In view of its topography and the histological structure of its wall (elastic artery, lack of ductal tissue), the initial segment of this type of isolated CCA can hardly be interpreted as the ipsilateral arterial duct. Moreover, in addition to the initial segment of the isolated LCCA, a remnant of the left-sided ductus arteriosus (Ligamentum arteriosum or stenotic vascular element with histologically verified ductal tissue) is identified at the bifurcation of the main pulmonary artery or at the origin of the left pulmonary artery [23, 29].

## Associated Malformations or Syndromes

The majority of cases with distal origin of the isolated CCA occurred in association with simple (e.g. VSD, ASD) or complex (e.g. ToF, DORV) congenital heart defects (16/20). Only three cases did not show any structural defect of the heart, and one case report did not provide information on associated congenital heart defects. A patent arterial duct connecting a pulmonary artery to the aortic arch was found contralateral to the isolated artery in 8 patients. If the initial segment of the isolated CCA is interpreted as the ipsilateral arterial duct, these 8 cases seem to show bilateral arterial ducts. The majority of cases with distal origin of the isolated CCA was furthermore characterized by the presence of extracardiac malformations or congenital syndromes (17/20). The most frequent syndrome was 22q11-deletion/DiGeorge-syndrome (8/20), followed by CHARGE-syndrome (5/20), while a third syndrome (Holt-Oram) was found only in a single patient. Only two patients did not show any cardiac as well as extracardiac malformation or congenital syndrome.

In contrast to the first subset, all previously published cases with proximal origin of an isolated LCCA had structurally normal hearts and did not occur in association with congenital syndromes. Extracardiac dysmorphologies were found only in two cases. These cases showed facial dysmorphologies such as hypertelorism with depressed nasal bridges [24] or mild hemifacial microsomia [29].

## Embryology

**Fig. 3:**
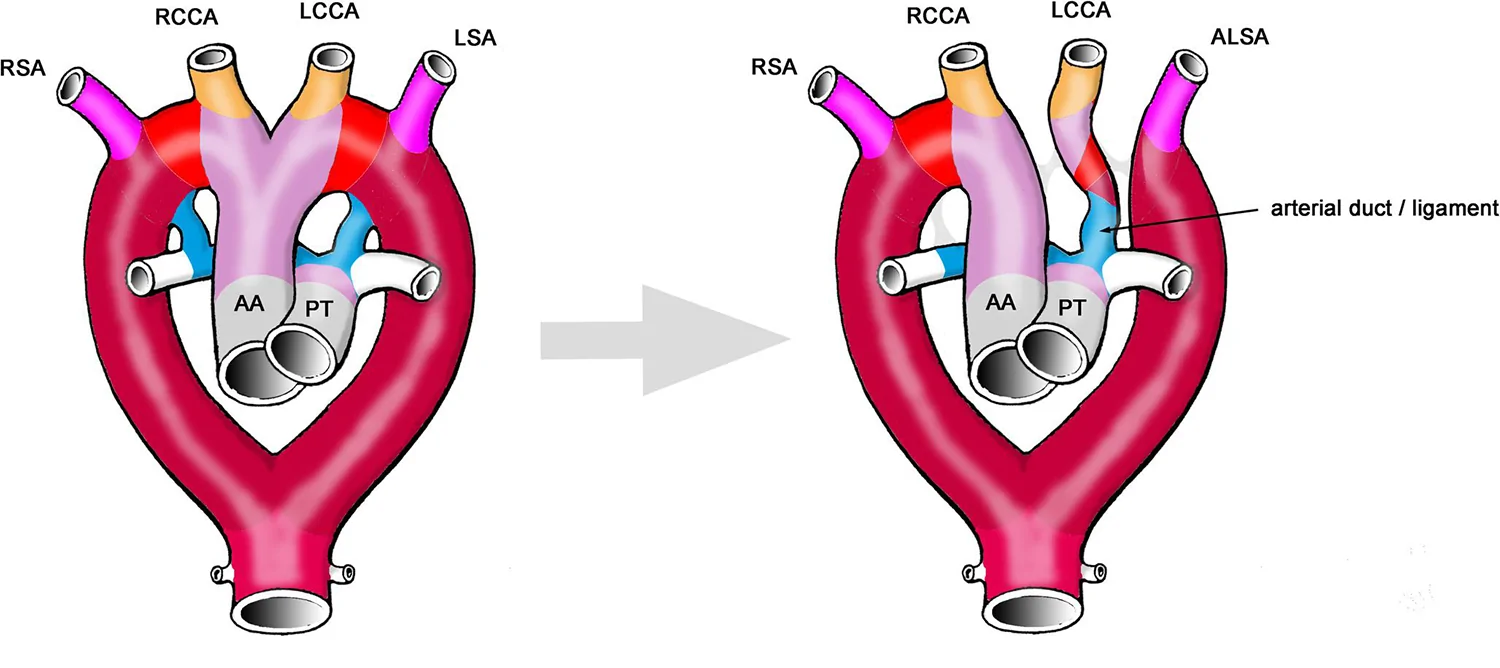
This drawing illustrates the pathogenesis of isolation of the left common carotid artery as explained by remodeling of the classical double aortic arch scheme of Edwards due to regression of arterial segments. The initial segment of the isolated artery is interpreted as derivative of the arterial duct. Abbreviation: RSA = right subclavian artery; other abbreviations as used in Figs. 1 + 2. Color code: orange = arterial segments derived from the 3rd pharyngeal arch arteries; red = arterial segments derived from the 4th pharyngeal arch arteries; blue = arterial segments derived from the so-called 6th pharyngeal arch arteries*; magenta = arterial segments derived from the 7th intersegmental arteries; grey = arterial segments derived from the distal outflow tract; purple = arterial segments derived from the aortic sac; reddish brown = segments derived from the paired dorsal aortae. Redrawn and modified after [34]. *Please note that, for several reasons, some researchers prefer usage of the term *pulmonary arch* instead of 6th pharyngeal arch artery [35, 36]

The morphogenesis of variants and malformations of the aortic arch system is traditionally explained by remodeling of the embryonic pharyngeal arch arterial complex. Starting with the fundamental works of Rathke [31] and Boas [32], several authors have introduced schemes of this arterial complex to conceptualize the morphogenesis of normal as well as abnormal branching patterns of the aortic arch system. A popular and widely used scheme of this kind is the double aortic arch of Edwards [33, 34]. The classical morphogenetic concept depicted by such schemes explains the definitive pattern of the aortic arch system exclusively by regression and persistence of embryonic vascular segments. Consequently, isolation of an aortic arch artery is traditionally explained by regression of arterial segments proximal and distal to this artery whereas its connection to a pulmonary artery can be explained only by persistence of the ipsilateral arterial duct. Thus, the classical morphogenetic concept usually identifies the initial segment of an isolated CCA as the ipsilateral arterial duct (Fig. 3). With regard to the first subset of isolated CCA, this interpretation is in accord with the above-described topography and postnatal behavior (narrowing, closure) of the initial segment of the isolated artery.

**Fig. 4:**
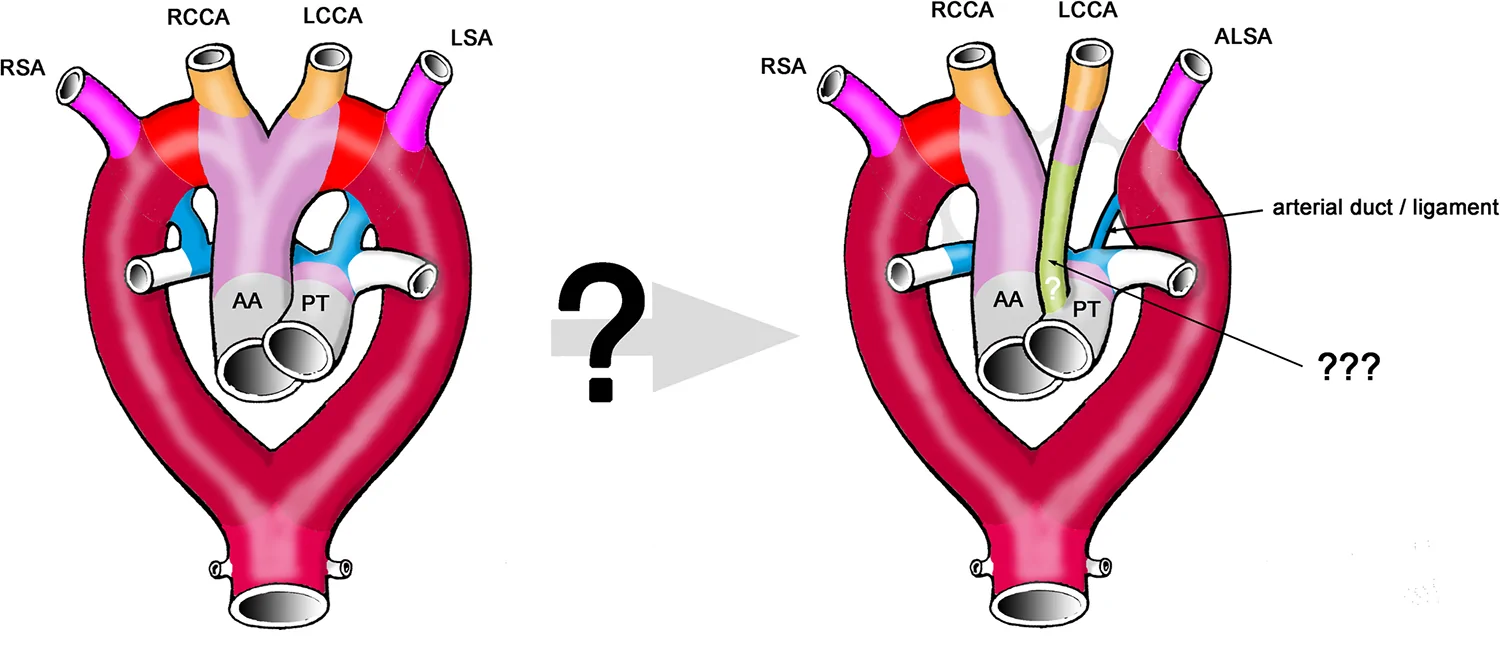
This drawing illustrates the fact that the classical concept of remodeling of the embryonic pharyngeal arch arterial complex cannot explain the second phenotype of isolation of the left common carotid artery. The ontogenetic identity of the initial segment of the isolated artery remains a mystery. Abbreviations and color code as used in Fig. 3

In contrast to the first type of isolated CCA, the initial segment of the second type of isolated CCA cannot be interpreted as the ipsilateral arterial duct (Fig. 4). Two questions arise: (1) what is the ontogenetic identity of the initial segment of this type of isolated CCA? (2) How became the isolated CCA separated from the developing aortic arch and connected to the proximal portion of the pulmonary trunk? The classical morphogenetic concept cannot answer these questions. The second phenotype of isolated CCA obviously is a challenge for a renowned embryological concept. Its existence suggest that, besides regression and persistence of embryonic arterial segments, there must exist a least a second mechanism involved in normal and abnormal remodeling of the embryonic pharyngeal arch arterial complex.

**Fig. 5:**
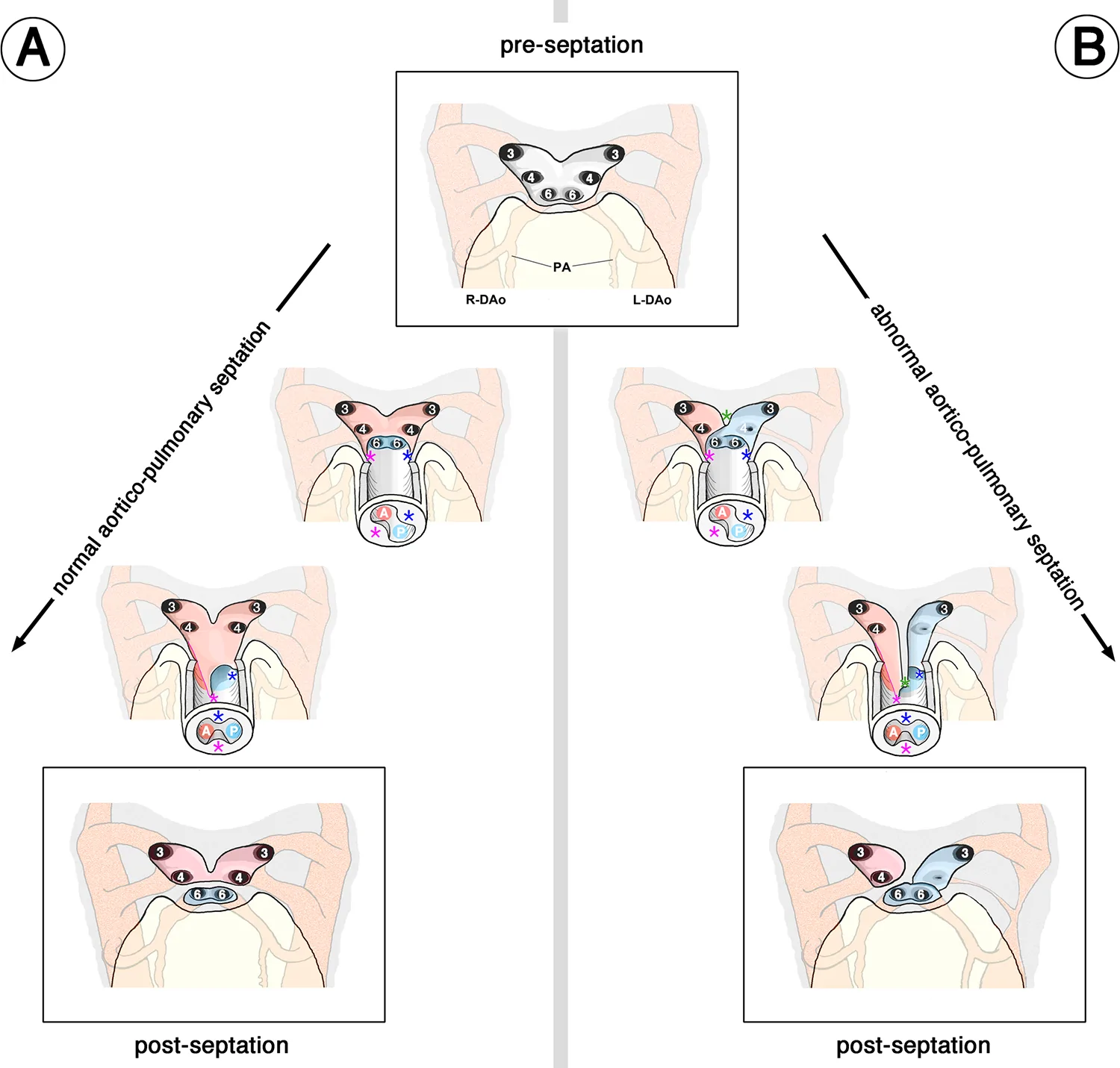
These semi-schematic drawings illustrate the process of septation of the aortic sac and distal outflow tract during normal embryonic development (**A**), and a hypothetical scenario leading to isolation of the left common carotid artery (**B**). Drawings depict ventral (downstream) views into the opened aortic sac and distal outflow tract. Arteries are depicted within the semitransparent parapharyngeal mesenchyme. Surfaces of the pericardial cavity are depicted in yellow color. **A**: During normal development, the paired flow dividers between the orifices of the 4th and 6th pharyngeal arch arteries unite and form the aortico-pulmonary (AP) septum, which growths upstream and thereby leads to the structural division of the aortic sac and distal outflow tract into aortic (reddish color) and pulmonary (bluish color) components. During upstream growth of the AP-septum, the distal outflow tract undergoes anti-clockwise torsion. The right and left spurs of the AP-septum (marked by red and blue asterisks) unite with paired endocardial ridges of the outflow tract (marked by corresponding red and blue asterisks). **B**: In the hypothetical scenario, the right-sided primordium of the AP-septum unites with an abnormally enlarged intercarotid flow divider (green asterisk). Thereby an irregular “AP”-septum is formed. This septum growths upstream and leads to an abnormal structural division of the aortic sac and distal outflow tract. The left-sided 3rd and 4th pharyngeal arch arteries now are separated from the aortic component (reddish color) and take an abnormal origin from the pulmonary component (bluish color). Regression of the left 4th pharyngeal arch artery and its adjoining segments of the left dorsal aorta complete isolation of the LCCA from the future aortic arch and its branches. Abbreviations: 3 = orifice of the 3rd pharyngeal arch artery; 4 = orifice of the 4th pharyngeal arch artery; 6 = orifice of the so-called 6th pharyngeal arch artery; A = flow path of the future ascending aorta; L-DAo = left dorsal aorta; P = flow path of the future pulmonary trunk; R-DAo = right dorsal aorta. Drawings in A are based on original image data from [41] (Fig. 33) and [42] (Figs. 3.1.43, 3.1.75) showing the normal aortic sac and pharyngeal arch arterial complex of human embryos at post-fertilization weeks 5 and 6

Data from studies on chick embryos suggest that, in the second phenotype of isolated CCA, the abnormal origin of the LCCA from the pulmonary trunk results from an abnormal septation of the aortic sac and distal outflow tract of the embryonic heart [37, 38]. The classical morphogenetic schemes focus on the remodeling of the pharyngeal arch arterial complex downstream to the aortic sac (extrapericardial arteries). They neglect the process of septation of the aortic sac and distal outflow tract that normally leads to the structural separation of the systemic and pulmonary arterial flow paths at the level of the intrapericardial arterial trunks (ascending aorta and pulmonary trunk). Structural separation of the systemic and pulmonary arterial flow paths upstream to the pharyngeal arch arteries is usually attributed to the upstream growth of a neural crest-derived flow divider named *aortico-pulmonary septum* (Fig. 5A). This septum arises from the union of initially paired flow dividers found between the orifices of the 4th and 6th pharyngeal arch arteries in the right and left halves of the aortic sac. The united septum forms a transverse ridge at the dorsal wall of the aortic sac from where it grows upstream and thereby divides the initially common aortic sac cavity into systemic (cranial) and pulmonary (caudal) components. Subsequent to structural division of the aortic sac, the aortico-pulmonary septum grows into the distal portion of the embryonic cardiac outflow tract where it leads to the structural separation of the systemic and pulmonary flow paths at the level of the future intrapericardial arterial trunks [39, 40].

The aortico-pulmonary septum is a well-known embryological structure. Largely unknown, however, is the fact that the aortico-pulmonary septum is not the only flow divider that forms within the aortic sac and contributes to vascular patterning and outflow tract septation of higher vertebrates. Anatomists from the Vienna University in Austria, such as Greil [43] and Pernkopf and Wirtinger [44], were the first to note this fact but their findings, as well as corresponding findings from other groups [45], did not receive much recognition during the past century. Besides the aortico-pulmonary septum, the Vienna anatomists describe two median flow dividers that might play a prominent role in the pathogenesis of the second phenotype of isolated CCA as well as other human aortic arch anomalies. One of these median flow dividers is located at the cranial pole and the other is located at the caudal pole of the aortic sac. They were named *intercarotid septum* and *interpulmonary septum*, respectively [44]. Recent works not only confirmed the presence of these median flow dividers [39, 46, 47]. They have furthermore confirmed that growth of these median ridges subdivides the initially common aortic sac cavity into left and right-sided sub-compartments [47]. In the systemic component of the aortic sac, these compartments are the left and right horns of the aortic sac, which normally become the brachiocephalic trunk and the initial part of the transverse aortic arch [48].

The malseptation concept postulates that an abnormal growth behavior of the intercarotid flow divider plays a fundamental role in the pathogenesis of the second phenotype of isolated CCA (Fig. 5B). With regard to the pathogenesis of isolation of the LCCA, an abnormally enlarged intercarotid flow divider is expected to unite with the right-sided primordium of the aortico-pulmonary septum so that an irregular “aortico-pulmonary” septum is formed. Upstream growths of such a septum would lead to an abnormal structural division of the aortic sac and distal outflow tract. The left-sided 3rd and 4th pharyngeal arch arteries now would be separated from the systemic component and take an abnormal origin from the pulmonary component. Regression of the left 4th pharyngeal arch artery and its adjoining segments of the left dorsal aorta would complete isolation of the LCCA from the future aortic arch and its branches.

This hypothetical concept identifies the initial segment of the second phenotype of isolated CCA as derivative of the distal portion of the embryonic outflow tract and aortic sac. This interpretation is in accord with several topographical and structural features of this arterial segment, such as (1) origin from the pulmonary circulation close to the root of the pulmonary trunk; (2) intrapericardial course along the ascending aorta where portions of its wall can be fused to the aortic wall [29]; (3) presence of a muscular media with well-preserved elastic lamellae characteristic of an elastic artery [24]; (4) the peculiar observation that a coronary artery can originate from the basal portion of this vascular segment [24].

The above-presented hypothetical scenario of aortic sac malseptation might lead not only to the second phenotype of isolation of a CCA but also to other rare congenital malformations of the aortic arch system such as isolation of the brachiocephalic (innominate) artery and type C interruption of the aortic arch [37]. A short review of previously published data on cases of isolated brachiocephalic artery indeed disclosed a phenotype in which the isolated artery took its origin from the proximal portion of the pulmonary trunk. Among the 86 previously published cases of isolated brachiocephalic artery, however, presence of this phenotype could be confirmed only in a single case [49]. The majority of available image data showed a distal origin of the isolated artery suggesting that malseptation of the aortic sac and outflow tract did not play a prominent pathogenetic role among previous cases of isolation of a brachiocephalic artery. It may be of interest, however, that the above-mentioned single case did not show any associated intracardiac defects or a congenital syndrome. The other phenotype of isolated brachiocephalic artery, however, frequently is associated with congenital heart defects and syndromes (DiGeorge, CHARGE, Goldenhar, Down, Heterotaxy).

**Fig. 6:**
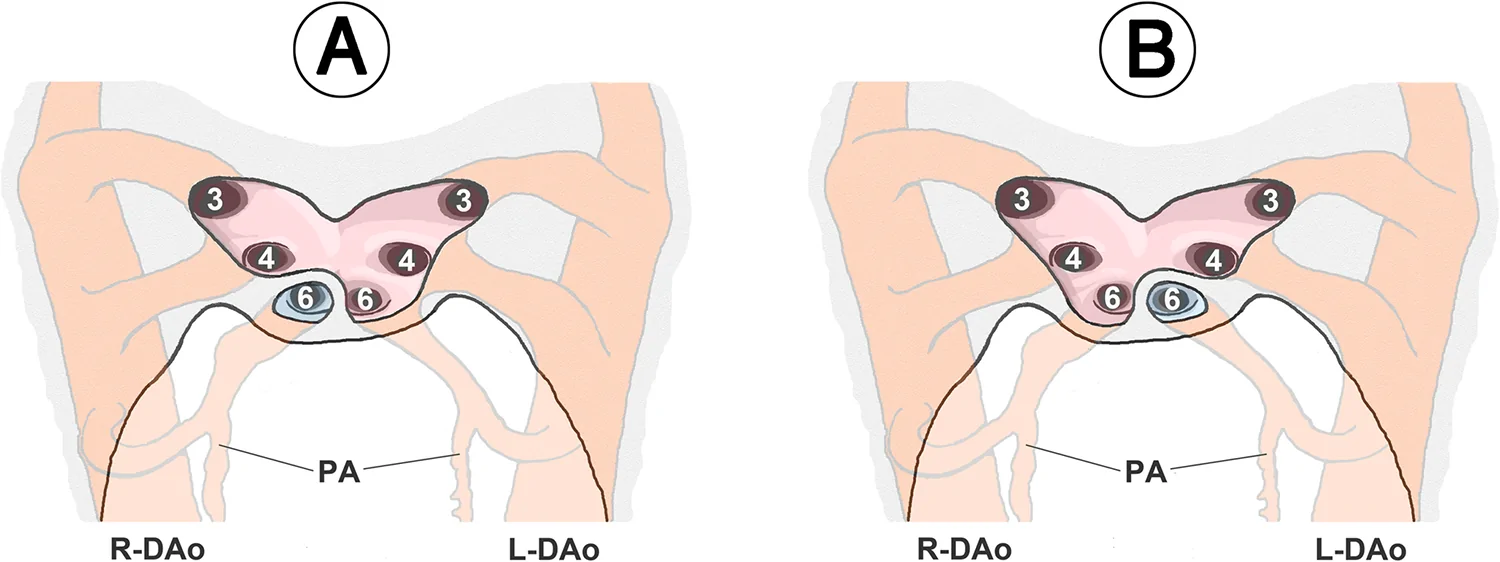
These semi-schematic drawings illustrate hypothetical patterns of abnormal aortic sac septation leading to abnormal origin of the left (**A**) and right (**B**) pulmonary artery from the ascending aorta. In these scenarios, an abnormal aortico-pulmonary septum is formed by union of the interpulmonary flow divider with the right (**A**) or left (**B**) aortico-pulmonary flow dividers. Abbreviations as used in Fig. 5

Malseptation of the aortic sac and distal outflow tract of the embryonic heart might not only lead to abnormal connections of systemic arteries to the pulmonary arterial tree, it might also lead to an abnormal origin of a pulmonary artery from the ascending aorta [50, 51]. Figure 6 illustrates hypothetical patterns of malseptation of the aortic sac that can explain the pathogenesis of this kind of vascular malformations. Future studies are needed to clarify the role of malseptation in the pathogenesis of congenital malformations of the aortic arch system.

I should finally make a comment on the fact that the majority of previously published cases of isolated CCA were cases of isolated LCCA and therefore occurred in association with a right aortic arch and aberrant left subclavian artery (Tables 1 and 2). It is well known that a right aortic arch rarely occurs in the general population but very often co-exists with congenital heart defects and malformation syndromes that affect the cardiac outflow (so-called cono-truncal malformations; DiGeorge syndrome, …) or lateralization of the inner organs (heterotaxy syndrome) [52]. The pattern of associated cardiac malformations and syndromes found among cases of the first phenotype of isolated CCA fits very well to the previous epidemiological data on the occurrence of a right aortic arch. The fact, however, that all of the previously published cases of the second phenotype of isolated LCCA showed structurally normal hearts and were not associated with syndromes does not fit to previous epidemiological data. Clarification of the situation awaits future studies.

## Data Availability

This review article does not report on new data. All original data are available and documented in the previously published case reports listed in Tables 1 and 2, as well as in References.

## Declarations

### Clinical Trial Registration

Not applicable. This study/review did not analyze new data from human patients.
